# ERP and Behavioral Effects of Physical and Cognitive Training on Working Memory in Aging: A Randomized Controlled Study

**DOI:** 10.1155/2018/3454835

**Published:** 2018-03-28

**Authors:** Patrick D. Gajewski, Michael Falkenstein

**Affiliations:** ^1^Leibniz Research Centre for Working Environment and Human Factors (IfADo), Dortmund, Germany; ^2^Institute for Working Learning Ageing (ALA), Bochum, Germany

## Abstract

Working memory (WM) performance decreases with age. A promising method to improve WM is physical or cognitive training. The present randomized controlled study is aimed at evaluating the effects of different training methods on WM. A sample of 141 healthy older adults (mean age 70 years) was assigned to one of four groups: physical training, cognitive training, a social control group, and a no-contact control group. The participants trained for four months. Before and after the training, *n*-back task during an EEG recording was applied. The results show that cognitive training enhanced the target detection rate in the 2-back task. This was corroborated by an increased number of repeated digits in the backward digit-span test but not in other memory tests. The improvement of WM was supported by an increased P3a prior to a correct target and an increased P3b both in nontarget and target trials. No ERP effects in the physical and no-contact control groups were found, while a reduction of P3a and P3b was found in the social control group. Thus, cognitive training enhances frontal and parietal processing related to the maintenance of a stored stimulus for subsequent matching with an upcoming stimulus and increases allocation of cognitive resources. These results indicate that multidomain cognitive training may increase WM capacity and neuronal activity in older age.

## 1. Introduction

Working memory (WM) reflects a complex cognitive function with limited capacity related to storage, update, and manipulation of contents enabling goal-directed behaviour [1]. A critical attempt in aging research is to find methods to improve WM capacity, as the basic executive function in elderly to preserve their quality of life. The most promising method to reduce age-related decline in working memory is practice [2]. In particular, formal cognitive training (CT) has been used in the last decade to enhance WM in the elderly (for reviews, [3–5]). Also, studies evaluating effects of physical activity (PA) on WM show positive effects (for reviews, [6–8]). A recent review by Ballesteros et al. [9] provides a comprehensive overview over theories of cognitive aging and neuroplasticity and different methods like CT, PA, dancing, and social engagement to enhance the neuroplasticity in older age. Also, Bamidis et al. [10] summarize several nonpharmaceutical interventions enhancing cognitive performance and preventing age-related cognitive decline. Moreover, they describe neurobiological mechanisms underlying the corresponding behavioral changes. In case of PA, neuronal plasticity is thought to be induced by the upregulation of neurotrophins enhancing neurogenesis and synaptic plasticity in the hippocampus and frontal brain areas. The functional mechanisms underlying CT benefits have been related to increased brain volume, cortical thickness, and increased coherence of white matter tracts. Additionally, Bamidis et al. [11] provided evidence that combined physical and cognitive activities result in even larger benefits than each intervention alone, suggesting synergistic effects of PA and CT that enhance functionally beneficial plastic changes to improve cognitive functions [10]. A meta-analysis of 23 PA and 21 CT randomized controlled studies compared effects of CT and PA on executive functions in older age and supported the view that both interventions improved executive functions [12].

On the downside, the results of training studies are inconsistent due to different measures, training contents, training duration, and intensity. In particular, studies comparing effects of CT and PA on executive functions or WM using the same measures are scarce [11, 13]. Thus, the efficiency of such interventions can only be compared using qualitatively different training methods with the same intensity and duration. Moreover, in case of positive effects of training reflected in performance, the electrophysiological underpinnings of these changes are less understood. Secondly, the lack of an adequate control group is often a problem in training studies and the reported training effects may be confounded by effects of new situations, devotion of trainers, and social aspects of training.

To compare effects of different training regimes and to avoid problems with an appropriate control, we conducted a randomized training study with two training and two control groups. Performance after cognitive and physical training was compared with a social control group and a no-contact control group. The social control group received a less demanding mild relaxation training that was not assumed to affect cognitive functions. In this way, effects of relaxation and social aspects of training were assessed.

The present study evaluated training effects on WM using a well-established *n*-back task consisting of a two-choice condition with low WM-load (0-back) and 2-back condition with high WM-load. In the *n*-back, task participants are presented a long sequence of visual stimuli and they are asked to determine for each stimulus whether it matches a stimulus *n*-trials before. In a 2-back task, for example, participants have to decide whether the current stimulus is the same as in the trial *n*-2. The *n*-back task was assumed to be a measure of WM capacity since it requires maintaining, continuous updating, and processing of information [14]. WM capacity refers to individual differences in performance in this task. It has been shown that the 2-back task is correlated with a complex span, different updating tasks, and fluid intelligence but not response-conflict [15, 16]. Our correlational analyses across 36 young and 36 old participants show an association between the 2-back performance and the digit-span backward, d2 and the word fluency task (unpublished data), supporting the idea that this task is a measure of working memory, attention, and speed of processing as well as fluid intelligence.

The *n*-back task shows age-related changes [17, 18]. In a previous study in our lab, performance and ERP activity were compared between young (mean age 25 years) and old (mean age 70 years) participants [19] (see also [20] for comparison between young and middle-aged groups). Older age was associated with slower responses and reduced rate of detected targets compared to the younger group in the 2-back condition. The corresponding ERP data showed a reduced P3a in the 2-back condition and a general reduction in the P3b in the older versus the younger subjects. The P3a has been related to stimulus-driven attentional processes and novelty processing [21–23]. In a more holistic model, P3a has been related to dopamine-driven working memory process [24]. The P3b was related to subsequent memory processes and allocation of cognitive resources to the task. Together, the frontoparietal P3a/P3b activity pattern is crucial for WM [25–27] and seems to reflect the transfer of stimulus information from the frontal (P3a) to temporal and parietal (P3b) brain areas [24].

Therefore, in addition to the training-related changes of performance in the *n*-back task, ERP was analysed in the present study. The few previous training studies evaluating ERP used different paradigms to assess training-induced changes. For example, Gajewski and Falkenstein and Gajewski et al.[28, 29], Küper et al. [30], and more recently Olfers and Band [31] conducted a task-switching paradigm measuring executive functions and cognitive flexibility and found an increased N2 potential associated with improved response selection after cognitive training. The results regarding the P3b related to enhanced allocation of cognitive resources were less consistent, whereas Gajewski and Falkenstein [28] found an increase but Gajewski et al. and Olfers and Band [29, 31] did not. This may be due to different types of training and age of the participants in these studies. A recent study by Tsai et al. [32] investigated the effects of different six-month long physical exercise interventions in elderly subjects and applied task switching as well as *n*-back tasks and measured ERP. They found faster responses during task switching and, more important regarding the present context, an improved target detection rate in the 2-back condition that was accompanied by an increased frontal and parietal P3 in the training compared to the control group.

As training studies in aging using ERPs are scarce and to the best of our knowledge training studies using *n*-back task and ERP assessing effects of cognitive training do not exist, specific hypotheses regarding particular components are elusive. Thus, comparison between ERP parameters in young and old adults is more suitable in order to formulate expected changes, assuming that enhanced training-related performance and brain activity in elderly shifts towards performance and brain activity of younger adults (rejuvenation).

Thus, we expect that training enhances the neuronal processing of update, storage, and maintenance involved in the 2-back task. This should be evident in enhanced performance, that is, more detected targets and/or faster responses to targets. These performance benefits should be accompanied by more pronounced neuronal processing. In terms of rejuvenation, we expect an increase in P3a and P3b after training that have been shown previously to be reduced in older as compared to young adults.

## 2. Methods

### 2.1. Participants

For a detailed description of the acquisition procedure and the characteristics of the sample, the reader is referred to [28, 33]. Briefly, the participants were healthy seniors. They were 65 to 88 years old (mean 70.1 years). They were included in the study after meeting a number of criteria inquired about by a telephone interview. They should be physically and mentally fit without any history of neurological, psychiatric, motor, cardiovascular or oncologic diseases, and not taking any psychopharmacological medication. No participants were included in the study if they already train physically (jogging, walking, swimming, dancing, and fitness centre) or cognitively (e.g., memory training) more than 1.5 hours weekly. A total of 467 telephone interviews were completed, 152 persons met the criteria, and were included in the study. Eleven participants dropped out during the study. Consequently, 141 participants constituted the final sample (see Table 1 in [28]).

The study was carried out in accordance with the Declaration of Helsinki and with the recommendations of the local ethics committee of the Leibniz Association. All participants gave written informed consent and received €100 to recompense them for travel expenses.

### 2.2. Training

Participants were randomly assigned to one of four groups: physical training group (PTG), cognitive training group (CTG), relaxation training group, (RTG) and a control group (CG). Participants were trained for 4 months, two times per week and 90 minutes per session. All training regimes were supervised by professional trainers. The reader is referred to [28, 33] for more detailed description of the training regimes.

Physical training (PT) consisted of cardiovascular, aerobic, and strength exercises. The cardiovascular training was conducted using treadmills, bicycle ergometer, and cross trainer which included pulse meter in order to monitor the heart function. The aerobic exercises consisted of a number of easy step and floor movement sequences. The muscular strength exercises were conducted using strength machines. These exercises were aimed at strengthening skeletal muscles and increasing metabolism. Intensity of the training units was continuously increased but considered the individual capability of the participants.

Cognitive training (CT) included paper and pencil and PC-based noncommercial exercises. In the first four weeks, a mental activation training (MAT [34]) was used. The MAT is a paper and pencil set with short exercises to increase working memory capacity, visual attention, and speed of information processing. Additionally, in the first eight sessions, participants without any PC experience were gradually made familiar with computer handling. In the following weeks, the participants exercised using selected commercial and noncommercial internet-based software (http://www.mentaga.com; https://www.ahano.de; https://www.mental-aktiv.de; https://www.freshminder.de). Each session consisted of different exercises that aimed at training crucial cognitive functions. The exercises were relatively complex and involved mainly attentional and mnemonic functions, but some exercises included multitasking or logical thinking and executive functions. Two extra sessions were offered at the end of the program for those participants who missed the regular sessions. The participants were not encouraged to exercise outside the training sessions.

The social control group (relaxation training group (RTG)) received relaxation training consisting of progressive muscle relaxation, autogenic training, back training, breathing exercises, massage, and Qigong. The aim of this training was to provide interesting and varied exercises which did not require, and hence should not train, complex cognitive functions such as working memory.

### 2.3. Neuropsychological Assessment

The test battery for measuring memory functions consisted of Verbal Learning and Memory Test (VLMT), a German version of the Auditory-Verbal Learning Test (AVLT), examining immediate verbal memory and delayed recognition of words. Recall from long-term semantic memory was measured by the Word Fluency Test (WFT) and short-term memory by the Digit-Span Test. Visuospatial memory was assessed by the Rey-Osterrieth Complex Figure (ROCF). Working memory was evaluated by the backward Digit-Span Test.

### 2.4. Stimuli and Procedure of the *n*-Back Task

Working memory was assessed using standardized 2-back task with a 0-back task as nonworking memory control condition in two separate blocks applied in the same order (0-back and 2-back) (see [14, 35] for all details of the task). The stimuli consisted of 25 capital letters which were presented centrally in white on a black computer screen for 300 ms. A fixation point was presented before each stimulus which was also located in the centre of the monitor (17 inches, refresh rate: 100 Hz, resolution: 640 × 480 pixels). In both blocks, the interstimulus interval (ISI-time) was 1500 ms, regardless whether the participant pressed a key or not.

In the 0-back block, a two-choice decision was required by pressing a response button for the target letter X and not responding when other letters occurred. In the 2-back task, the participants were asked to press a response button if the current letter was the same as the letter which was presented two trials before (target). Each block consisted of 20% target and 80% nontarget letters. The 2-back block included 156 trials while the 0-back block included 102 trials. The participants were given written instructions explaining the task. The instructions encouraged responding quickly and accurately.

### 2.5. ERP Recordings

EEG was recorded continuously from 32 scalp electrodes according to the extended 10–20 system mounted on an elastic cap. The montage included 8 midline sites and 12 sites on each hemisphere and two mastoid electrodes (M1 and M2). The EEG was rereferenced offline to averaged mastoids. The horizontal and vertical EOG was recorded bipolarly from electrodes at both eyes. Eye movement artefacts were corrected using the correction algorithm of Gratton et al. [36]. Electrode impedance was kept below 10 kΩ. The amplifier bandpass was 0.01–140 Hz. EEG and EOG were sampled continuously with a rate of 2048 Hz. Offline, the EEG was downscaled to a sampling rate of 1000 Hz and cut in stimulus- and response-locked epochs by using the software Brain Vision Analyzer (Brain Products, Munich). Epochs in which the amplitude exceeded ±150 *μ*V were rejected. The ERPs were filtered digitally offline with a 10 Hz low and 0.05 Hz high pass.

### 2.6. ERP Waveform Analysis

The EEG was subdivided into epochs of the length 2000 ms, starting 500 ms before and ending 1500 ms after the stimulus onset defined as the time point of the pulse from the simulation computer. Peak amplitudes and latencies were measured automatically. In order to avoid boundary hits, only local maxima or minima within the interval were considered.

The P3a and P3b amplitudes were quantified as maximum amplitude in the time range between 300 and 600 ms at Fz (P3a) and Pz (P3b). The P3a and P3b amplitudes were analyzed at the same positions as in the previous studies to ensure comparability with previous results [19, 23, 24].

### 2.7. Statistical Analysis

For the analysis of behavioural data, maximum reaction time (RT) of 1500 ms and a minimum RT of 100 ms were allowed; otherwise, this response was categorized as missing. Only correct responses in the RT analysis were considered. A repeated measure analysis of variance (ANOVA) was conducted to compare the effect of group (physical (PTG), cognitive (CTG), relaxation (RTG), control (CG)), task type (0-back versus 2-back), and session (*t*1 versus *t*2) on RT and the ratio of missed targets.

Results of the neuropsychological tests were submitted to repeated measures ANOVA with the factor group (PTG, CTG, RTG, and CG) and session (*t*1 versus *t*2).

Regarding ERPs, in order to reduce complexity of the data, target-locked and nontarget-locked ERPs were analysed separately. Additionally, both trial types differed in the motor requirements (no response in nontargets and a response in target trials) making the data not directly comparable. Moreover, specific differences between these conditions were not in focus of the present study and were analysed elsewhere (for corresponding analyses see [19]).

The ANOVA design for the analysis of the ERP data included the between-subject factor group (PTG, CTG, RTG, and CG), within-subject factors task type (0-back versus 2-back), and session (*t*1 versus *t*2) at particular a priori selected electrodes (see above). In order to identify the origin of training-induced changes in more detail, a separate analysis for the P3a and P3b was conducted for nontarget trials prior to a correctly detected target in trials *n*-2 and *n*-1. This allows evaluation of encoding of an item (trial *n*-2) and maintaining of this item in working memory for later use (*n*-1). The corresponding ANOVA design included the factors trial sequence (*n*-2 versus *n*-1), task type (0-back versus 2-back), and group (PTG, CTG, RTG, and CG).

To resolve specific effects or interactions, Bonferroni-corrected follow-up ANOVAs were conducted. Finally, to assess group differences after training difference scores (*t*1-*t*2) were computed and a priori contrasts were conducted (1) PTG versus RTG, (2) PTG versus CG, (3) CTG versus RTG, (4) CTG versus CG, and (5) PTG versus CTG. The statistical analysis was conducted by IBM SPSS 23.0.

## 3. Results

### 3.1. Neuropsychological Data

Different aspects of memory were evaluated using several neuropsychological tests. The verbal short- and long-term memory as well as delayed recognition of words assessed by VLMT revealed a general improvement over time but no single one interaction group × session was significant. Recall from long-term semantic memory assessed by the Verbal-Fluency-Test showed a weak trend for the interaction group × session (*F*(3, 137) = 2.17, *p* = 0.094), suggesting a slightly increased number of produced words at *t*2 (49.3) than *t*1 (44.4) in the CTG group, whereas no changes were observed in the remaining groups (PTG: *t*1 (43.6) versus *t*2 (44.2), RTG: *t*1 (39.4) versus *t*2 (38.8), CG: *t*1 (42.6) versus *t*2 (43.9). The delayed recall performance of the complex Rey-Osterrieth figure showed an effect of session (*F*(1.137) = 63.02, *p* < 0.0001), suggesting better performance at *t*2 in all groups but did not reveal any specific improvements due to training (*F*(3, 137) = 1.23, *p* = 0.3). Immediate recall, that is, short-term memory assessed by digit span forward showed an effect of session (*F*(1.137) = 35.42, *p* < 0.0001) and no interaction session × group (*F* < 1). In contrast, and most importantly, working memory evaluated by the digit-span backwards showed an effect of session (*F*(1, 135) = 75.21, *p* < 0.0001) and an interaction session × group (*F*(3, 135) = 2.77, *p* = 0.044), suggesting an increase in the number of correctly repeated digits backwards in the CTG at *t*2 (7.5) than *t*1 (5.8), whereas less changes were observed in the other groups (PTG: *t*1 (5.8) versus *t*2 (6.6), RTG: *t*1 (5.4) versus *t*2 (7.2), CG: *t*1 (5.7) versus *t*2 (6.5)). A priori contrasts revealed strong trends for the comparisons CTG versus CG (*t*(135) = 1.95, *p* = 0.054) and CTG versus PTG (*t*(135) = 1.92, *p* = 0.057).

In sum, several memory parameters improved from *t*1 to *t*2 but no influence of training on short- or long-term memory was found. The only exception was the digit-span test backwards assessing working memory that showed improved performance in the CTQ relative to the PTG and CG groups.

### 3.2. Behavioral Data

The ANOVA on RTs revealed the main effect of session (*F*(1.137) = 6.4, *p* = 0.013), suggesting faster responses at *t2* than *t1,* an effect of task (*F*(1.137) = 741.9, *p* < 0.0001), indicating slower responses in the 2-back than 0-back task and an interaction session × task (*F*(1.137) = 8.4, *p* < 0.005) that documents faster responses at *t2* than at *t1* in the 2-back task (620 versus 642 ms), while no difference was found for the 0-back task (467 versus 467 ms). Despite the fact that the interaction session × task × group was nonsignificant, the groups differently contributed to the reduction of RTs in the 2 back task between *t1* and *t2*, whereas the physical training group significantly reduced the RTs in the 2-back condition from *t1* to *t2* (660 versus 623 ms; *F*(1.34) = 11.5, *p* < 0.005), no differences between sessions were found in the remaining three groups (all *F*'s < 1). No further effects or interactions reached significance. A priori contrasts for the difference between pre- and postmeasure in RTs in the 2-back task did not yield any differences (all *p*'s > 0.05).

Regarding the ratio of missed targets as a measure of working memory capacity, the ANOVA showed an effect of session (*F*(1.137) = 17.0, *p* < 0.0001), suggesting a reduction of omitted targets from *t1* to *t2* from 9.5 to 6.5%. As expected, the ratio of missed targets was larger in the 2-back than 0-back task, resulting in a main effect of task (*F*(1.137) = 193.4, *p* < 0.0001). Moreover, session and task interacted significantly (*F*(1.137) = 16.8, *p* < 0.0001), indicating reduced target omission ratio in the 2-back task between *t*1 and *t*2 (18.9 versus 13.0%), while no difference was observed for the 0-back task (0.2 versus 0.1%). The interaction session × group did not reach significance (*F*(3, 137) = 1.8, *p* = 0.14) and the second-order interaction session × task × group revealed only a trend (*F*(3, 137) = 2.1, *p* = 0.09). This trend was due to different performance benefits in the 2-back condition between *t*1 and *t*2 across the groups, while the physical group (15.7 versus 13.9%; *F*(1.34) = 1.1, *p* = 0.29), the relaxation (20.6 versus 15.3%, *F*(1.33) = 3.3, *p* = 0.08), and the non-contact group (19.9 versus 15.1%; *F*(1.39) = 2.0, *p* = 0.16) did not or only marginally improve performance, the cognitive training group strongly reduced the ratio of missed targets from *t1* to *t2* (19.3 versus 8.8%; *F*(1.31) = 12.8, *p* < 0.001). A priori contrasts for the difference between pre- and postmeasure in target detection ratio in the 2-back condition revealed differences between CTG and CG (*t*(137) = 2.4, *p* = 0.016), a trend for the difference CTG versus RTG (*t*(137) = 1.8, *p* = 0.068), and a difference between PTG versus CTG (*t*(137) = 2.7, *p* = 0.007).

In sum, despite the fact that the interaction between the factors group and session did not reach significance, the physical training group improved their processing speed numerically at *t*2 versus *t*1, whereas the cognitive training group improved working memory capacity indicated by an enhanced accuracy in the 2-back condition at *t*2 versus *t*1. This improvement of performance in CTG was also corroborated by a significant difference between CTG and PTG and CTG and CG at *t*2 in the 2-back task.

### 3.3. ERP

#### 3.3.1. P3a

The mean amplitude of the P3a at Fz in *target trials* was larger in the 0-back than the 2-back condition (5.0 *μ*V versus 4.3 *μ*V; *F*(1.137) = 8.3, *p* < 0.005). Moreover, there was a trend of session (*F*(1.137)=3.6, *p* = 0.059), indicating an increase in the P3a at t2 (4.9 *μ*V) relative to *t*1 (4.3 *μ*V). No further effects or interactions reached significance.

**Figure 1:**
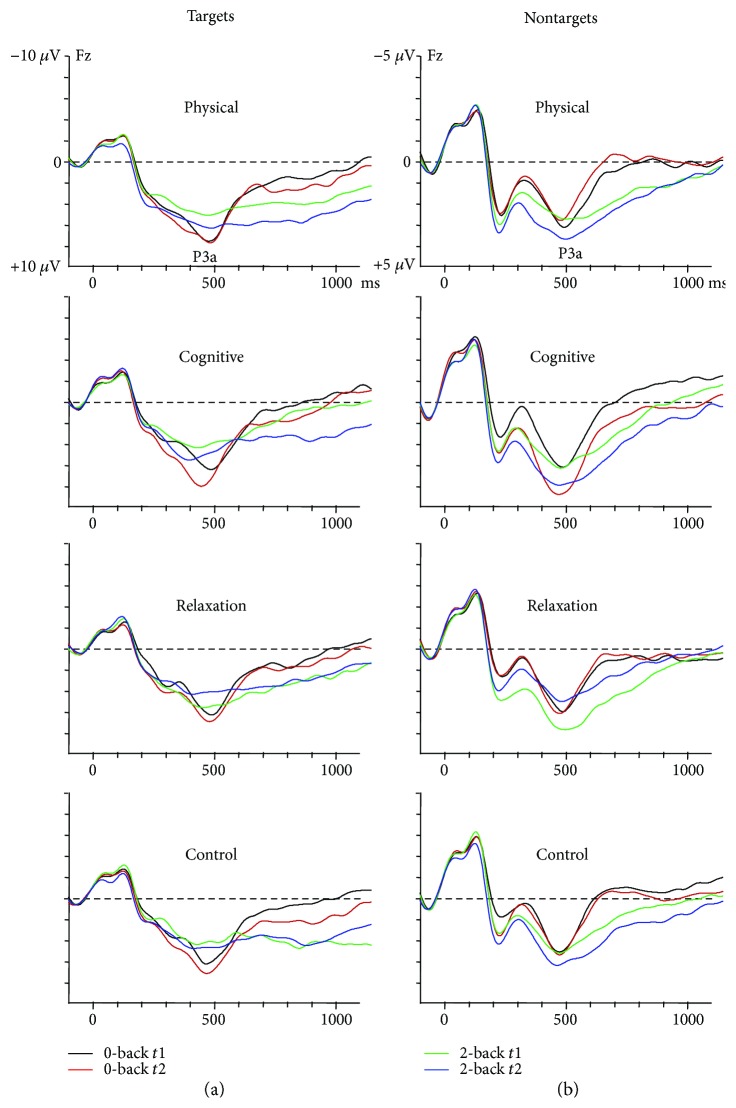
P3a in target (a) and nontarget (b) trials of the 0-back and 2-back task in the four groups. Note the different scaling of targets and nontargets.

The P3a amplitude in *nontarget trials* was more pronounced in the 2-back than 0-back task (2.4 *μ*V versus 1.7 *μ*V; *F*(1.137) = 21.0, *p* < 0.0001). There was no effect of session (*F*(1.137) = 2.6, *p* = 0.105) and a trend for session × group interaction (*F*(3, 137) = 2.3, *p* = 0.077). More importantly, however, was the interaction task × session × group (*F*(1.137) = 4.2, *p* < 0.01). In order to resolve this interaction, the effect of task and session was evaluated in each group separately. In the PTG, there was an effect of task due to larger P3a in 2-back than 0-back (2.5 versus 1.5 *μ*V; *F*(1.34) = 5.1, *p* < 0.05). Despite the apparently larger P3a at *t*2 than *t*1 in the 2-back condition (cf.  Figure 1), no effect of session or interaction task × session were found. In the CTG, no effect of task was obtained. However, an effect of session (*F*(1.30) = 4.9, *p* < 0.05) indicated a larger P3a at t2 than *t*1 (3.0 *μ*V versus 2.1 *μ*V). The interaction task × session did not reach significance. The RTG showed an effect of task (*F*(1.34) = 9.0, *p* < 0.005), due to a larger P3a in 2-back than 0-back (2.4 *μ*V versus 1.5 *μ*V), no effect of session and an interaction task × session (*F*(1.34) = 9.1, *p* < 0.005), showing a similar amplitude in the 0-back task at *t*1 and *t*2 (1.3 *μ*V versus 1.6 *μ*V) and an amplitude reduction in *t*2 versus *t*1 in the 2-back task (2.7 *μ*V versus 1.7 *μ*V). Finally, the CG showed merely an effect of task on the P3a (*F*(1.39) = 17.6, *p* < 0.0001), being larger in 2-back than 0-back (2.0 *μ*V versus 1.3 *μ*V). The effect of session and the interaction session × task were not significant. A priori contrasts for the difference between pre- and postmeasure in the 2-back condition revealed an increase in the P3a in CTG relative to RTG (*t*(137) = 2.3, *p* = 0.006).

In sum, after training, the P3a was enhanced in the CTG and remained stable in the PTG. In contrast, in the RTG, the P3a was reduced after the training. No changes were found in the CG.

#### 3.3.2. Sequential Analysis of the Nontarget P3a

In order to identify the source of the training effect in more detail, we analysed the P3a amplitude in the last two nontarget trials preceding a target trial (*n*-2 and *n*-1) which are correspondingly related to the encoding (*n*-2) and maintenance (*n*-1) of the stimulus later used for the comparison with the target. The ANOVA revealed an effect of the factor trial sequence, showing a larger P3a in *n*-1 than *n*-2 (2.9 *μ*V versus 2.2 *μ*V; *F*(1.137) = 19.8, *p* < 0.0001), an effect of task (*F*(1.137) = 40.2, *p* < 0.0001), corroborating a larger P3a in 2-back than 0-back (3.2 *μ*V versus 1.9 *μ*V). Moreover, the interaction session × group (*F*(3, 137) = 3.5, *p* < 0.05) indicated a P3a increase in the CTG from *t*1 to *t*2 (2.3 *μ*V versus 3.7 *μ*V, *p* < 0.05), whereas no changes were obtained in the remaining groups (PTG: 2.5 *μ*V versus 2.5 *μ*V, RTG: 2.4 *μ*V versus 2.2 *μ*V, CG: 2.3 *μ*V versus 2.2 *μ*V, all *p*'s > 0.05).

**Figure 2:**
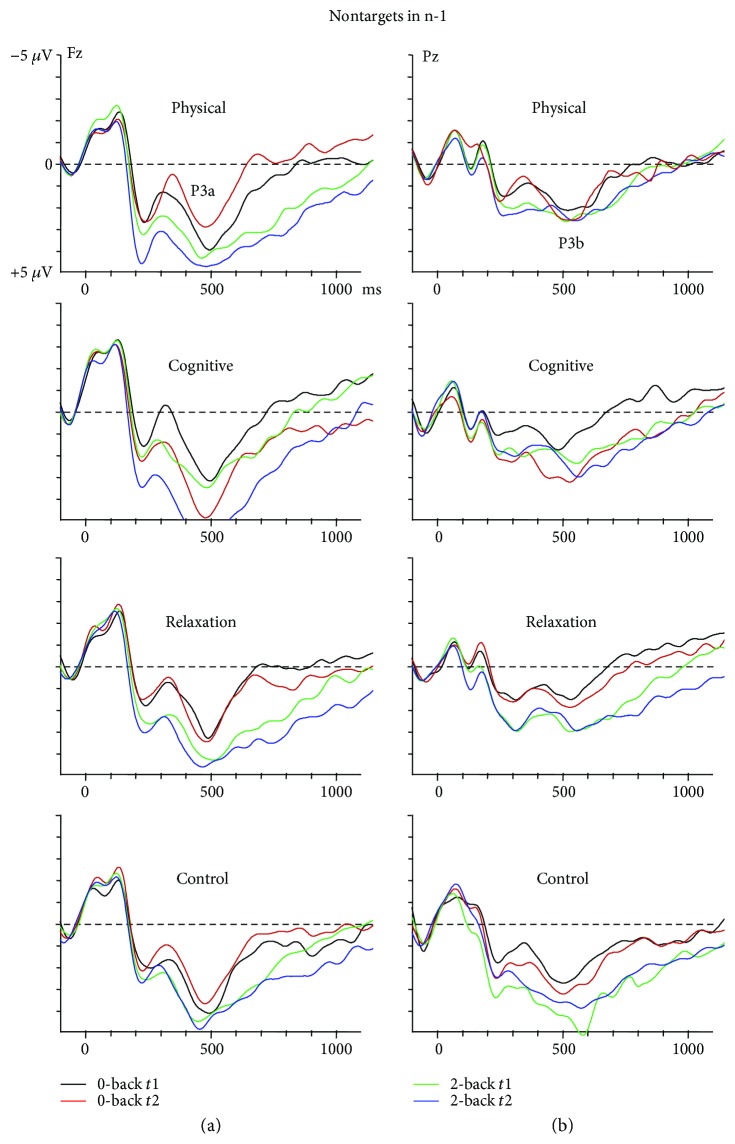
P3a (a) and P3b (b) in nontarget trials prior to correctly detected target trials (*n*-1) in the 0-back and 2-back task for the four groups.

In the next step, two ANOVAs were conducted separately for nontarget trials in *n*-2 and in *n*-1. In the trial *n*-2, only an effect of task was found (*F*(1.137) = 28.5, *p* < 0.0001). However, in the trial *n*-1, besides a task effect (*F*(1.137) = 24.8, *p* < 0.0001), an interaction session × group was confirmed (F(3, 137) = 3.2, *p* < 0.05).  Figure 2(b) illustrates this pattern. In order to resolve this interaction, ANOVAs including session × task were conducted for each group separately. The PTG showed larger P3a in *n*-1 in the 2-back than 0-back task (3.5 *μ*V versus 2.1 *μ*V; *F*(1.34) = 9.1, *p* < 0.005). No effect of session (*F* < 1) and a trend for the interaction task × session were seen (*F*(1.34) = 3.1, *p* = 0.085). In the CTG, there was a trend of task (*F*(1.30) = 3.3, *p* = 0.079) suggesting larger amplitude in the 2-back than 0-back (3.9 *μ*V versus 2.8 *μ*V) and more importantly an effect of session (*F*(1.30) = 6.6, *p* = 0.015), indicating an increase in the P3a from 2.5 *μ*V at *t*1 to 4.2 *μ*V at *t*2. The interaction was not significant (*F* < 1). The RTG and the CG showed only main effects of task with larger P3a amplitudes in the 2-back than the 0-back task, (RTG: *F*(1.34) = 7.7, *p* = 0.01; CG: *F*(1.39) = 5.6, *p* < 0.05). No further effects or interactions reached significance.

A priori contrasts for the difference between pre- and post- measure in the 2-back condition revealed an increase in the P3a immediately prior the target in CTG relative to CG (t(137) = 2.1, *p* = 0.035) and RTG (t(137) = 1.9, *p* = 0.056).

In sum, the training-related increase of the P3a in the CTG was only seen in non-targets in the trial *n*-1 preceding a correctly detected target and relative to the control groups.

#### 3.3.3. P3b

**Figure 3:**
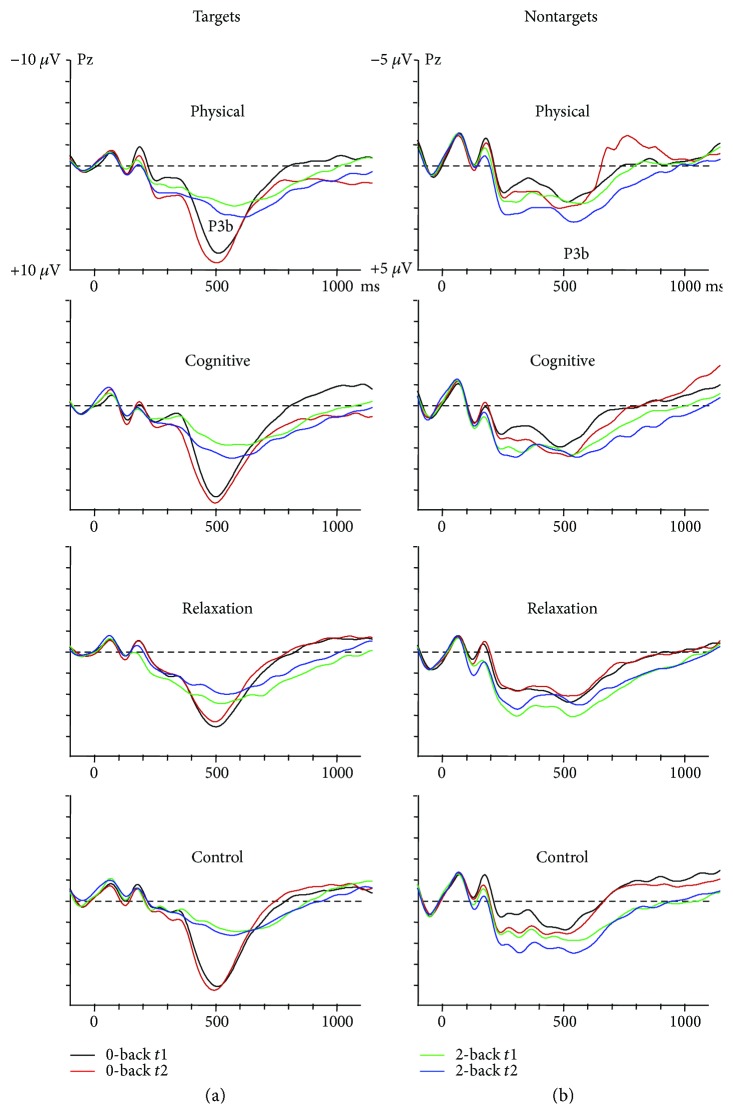
P3b in target (a) and nontarget (b) trials of the 0-back and 2-back task in the four groups. Note the different scaling of targets and non-targets.

Figure 3 shows the P3b in target and nontarget trials. The P3b amplitude in *target trials* at Pz showed an effect of task, suggesting a larger amplitude in 0-back than 2-back (5.0 *μ*V versus 2.9 *μ*V; *F*(1.137) = 63.0, *p* < 0.0001) and an interaction session × group (*F*(3, 137) = 4.2, *p* < 0.01). This interaction indicated a similar P3b at *t*1 and *t*2 in the PTG (2.5 *μ*V to 2.9 *μ*V, *p* = 0.423) and CG (2.0 *μ*V versus 2.5 *μ*V, *p* = 0.607), while there was an increase from *t*1 to *t*2 in the CTG (1.5 *μ*V versus 2.8 *μ*V, *p* = 0.012) and a decrease in the RTG (3.3 *μ*V versus 2.0 *μ*V, *p* = 0.036). A priori contrasts for the difference between pre- and postmeasure in the 2-back task revealed an increase of the P3b in the target in CTG relative to RTG (*t*(137) = 2.1, *p* = 0.024) and a trend for the difference PTG versus RTG (*t*(137) = 1.9, *p* = 0.073).

The mean P3b amplitude in *nontarget trials* was larger in the 2-back than 0-back task (0.9 *μ*V versus 0.3 *μ*V; *F*(1.137) = 12.7, *p* < 0.001). More importantly, there was an interaction task × session × group (*F*(3, 137) = 3.4, *p* < 0.05). In order to resolve this interaction, the effect of task and session was evaluated in each group separately. In the PTG, there was an effect of task due to larger P3b in 2-back than 0-back (1.3 *μ*V versus 0.2 *μ*V; *F*(1.34) = 4.4, *p* < 0.05). No effects of session (*F* < 1) or interaction task × session was found (*F*(1.34) = 2.7, *p* = 0.140). In the CTG, no effect of task or interaction task × session was obtained (both *F*'s < 1). However, an effect of session (*F*(1.30) = 4.4, *p* < 0.05) indicated a generally larger P3b at *t*2 (0.8 *μ*V) than *t*1 (0.3 *μ*V). The RTG showed an effect of task (*F*(1.34) = 15.4, *p* < 0.0001), due to larger P3b in 2-back than 0-back (1.0 *μ*V versus 0.3 *μ*V), no effect of session (*F*(1.34) = 1.9, *p* = 0.194), and an interaction task × session (*F*(1.34) = 10.9, *p* < 0.005), suggesting a similar amplitude in the 0-back task at *t*1 and *t*2 (0.3 *μ*V versus 0.3 *μ*V) and a reduction in the 2-back task (1.4 *μ*V versus 0.5 *μ*V). In the CG, only an effect of task was obtained (*F*(1.39) = 7.0, *p* < 0.05). No further effects or interactions were found. A priori contrasts for the difference between pre- and postmeasure in the 2-back task showed a trend for larger increase of the P3b in the PTG relative to the RTG group (*t*(137) = 1.8, *p* = 0.068). No further contrasts reached significance.

In sum, the P3b after target and nontargets increased from *t*1 to *t*2 in the CTG, remained stable in the PTG, and decreased in the RTG in the 2-back condition.

## 4. Discussion

The aim of the present study was to compare the effects of cognitive (CT) and physical (PT) training on different memory parameters and especially working memory (WM) compared to a social control group (RTG) and a no-contact control group (CG). Neuropsychological data showed a nonspecific improvement in all groups, while a specific effect of training was only found in the backward digit-span test in the CTG. This enhancement was evident relative to the CG and the PTG.

Although the interaction between the time point of the measurement (pre- versus post-) and group did not reach significance in the behavioral data of the *2*-back task, a priori contrasts showed an increased detection rate of targets in the CTG, suggesting improved WM capacity relative to the CG and PTG. This result exactly supports the finding of the backward digit-span test, suggesting an increase in WM capacity after cognitive training. The ERP data showed a number of changes from pre- to postmeasure: the amplitude of the nontarget P3a increased in the CTG, remained stable in the PTG and CG, and decreased in the RTG. The increase in the P3a in CTG was substantial relative to the RTG and CG. Thus, the used multidimensional cognitive training improved working memory capacity that was accompanied by an enhancement of the P3a. The ERP findings give some hints concerning the source of the behavioural effect. In particular, the separate analysis of target and nontarget trials as well as nontargets preceding a correct target in *n*-2 and *n*-1 allows identification of the neuronal origin of this effect in more detail. The fact that the P3a was not increased in target trials requiring the matching process between the upcoming stimulus with the stored item to select a correct response indicates that this is not the crucial process improved by the training. In contrast, the P3a increase in *n*-1 nontarget trials suggests that rather encoding or maintenance of the stimulus in WM was enhanced. In other words, the results indicate that the training effect was restricted to an amplitude increase in nontargets in the trial immediately preceding the correctly detected target. Therefore, it seems that a strengthened maintenance of the temporarily stored stimulus is the source of a higher target detection rate in the subsequent trial in the CTG group. Enhanced maintenance of stored items would also explain the result of the backward digit-span test as a stable representation of stored items and is necessary to manage the task.

In contrast to the P3a, the P3b was generally enhanced after nontargets as well as targets in CTG. This suggests that cognitive training also increases the general allocation of cognitive resources to the WM task. Moreover, the P3b in nontargets was larger in CTG than RTG in targets of the 2-back task. The increase in the P3b supports the findings obtained recently by Tsai et al. [32], and our earlier results obtained with a task switching task in the same subjects [28].

In contrast to the cognitive training group, the physical group (PTG) showed no session effects and no WM capacity improvements. Hence, the numeric decrease in target RTs seen after training cannot be attributed to changes in the P3 complex. Rather, a change of motor activation or threshold may be the reason for this trend. This should be investigated in further studies. Finally, the social control group received relaxation training that was used as an activity that has not been associated with improvements of cognitive functions. Indeed, in contrast to the physical and cognitive training group, no behavioral effects were obtained in the relaxation group. Moreover, the ERP pattern differed from the other groups by reduced amplitude and latency of the P3a and P3b relative to premeasure. Hence, it can be speculated that relaxation training is associated with less intense neuronal processing which should be investigated in further studies. The no-contact control group revealed no effects on behaviour and ERPs which shows that test repetition after a relatively long time period such as 4 months does not markedly change cognitive and neuronal processes. Thus, task repetition effects can be probably neglected when the distance between testing is long enough, as in the present study.

Taken together, improvements in working memory performance due to cognitive training were associated with an enhancement of both P3a and P3b. The P3a effect in nontarget trials was mainly present in trials preceding a correct target, suggesting enhanced maintenance of stored items to match it with the upcoming stimuli. The P3b increase was observed in target trials, suggesting gained allocation of cognitive resources to the task after CT.

The P3 complex seems to play an important role in the 2-back paradigm. In a previous study, performance and ERPs were compared between young and older adults [19]. Expectedly, older adults showed an impaired performance in speed and accuracy which was accompanied by a specific reduction in the P3a in the 2-back condition and a nonspecific reduction in the P3b relative to the young group. A similar reduction in the P3b was also found in low performers using the same task [35]. Therefore, cognitive training appears to induce rejuvenation and reduce the age-related decline by increasing both the P3a and the P3b that seems to be associated with WM capacity. This supports the idea that P3a and P3b activity reflects the transfer from frontal to parietal areas that enables WM operations [24].

The results of the present study are in line with results of a number of studies evaluating effects of computerized cognitive training on executive functions and WM capacity in the elderly [9, 10]. Most of them reported positive effects on cognition and brain plasticity [3, 4, 10, 37, 38]. However, some studies showed no or weak effects of video games on WM [39, 40]. Accordingly, a recent meta-analysis also provides evidence of modest effects of computerized training on WM [5]. This is apparently due to large heterogeneity and differences in training efficacy between studies, depending on cognitive domain, duration and intensity of the training, supervised or unsupervised at-home training, and type of training like action video games, non-action video games, or different measures and tasks targeting cognitive domains. Moreover, training success can be also related to varying difficulty of the exercises and adaptive versus nonadaptive levels of difficulty of the individual performance of the trainees. Thus, the large number of factors affecting the efficacy of CT may contribute to the inconsistent results. An optimal combination of these factors to reach an improvement of WM capacity based on the meta-analysis [5] suggests a group-based, multidomain training, with session length between 30 and 60 minutes and frequency of 2-3 sessions per week. The design of the present study met most of these criteria.

### 4.1. Limitations

One limitation of the study was the relatively rigorous selection criteria for participants. The 152 participants were selected from 467 older volunteers on the basis of good physical and cognitive functioning and no history of psychiatric, neurological, cardiovascular, or oncologic diseases. At the same time, exclusion criteria were regular physical or cognitive training. Thus, the sample was presumably not representative of an average population of the elderly.

Secondly, the training regimes consisted of a number of subtrainings, for example, a part of the cognitive training represented paper and pencil units, later a computer-based battery using selected exercises from different training packages. Therefore, the training effect on WM may be due to a number of factors included in a multidomain cognitive training that cannot be disentangled. Moreover, the lack of effects in the physical group was unexpected. We assume that firstly, the training intensity and the ratio of aerobic training were too low and secondly, the total duration of the intervention was too short to observe stable effects on cognitive functions and to induce neural plasticity.

Finally, the EEG data were digitally filtered using a 10 Hz low-pass filter. This may reduce the absolute amplitudes of the ERPs. The reason for using this low-pass filtering was to enhance the detectability of the P3a that was difficult to find in some individuals using a higher cut-off. As the same filter was used in our previous study [19], we preferred to use the same data analysis strategy to ensure comparability between the studies. Moreover, we did not use a trigger-box during EEG recording. Instead, trigger pulses via parallel port were sent from the stimulation computer to the EEG-system. This may sporadically lead to timing inaccuracies.

## 5. Conclusions

This study shows different effects of the three trainer-guided training regimes, which were each conducted for 4 months twice a week. The cognitive training led to an increased performance in a backward digit-span task and 2-back task compared to the control groups, indicating improvement of WM capacity. In the ERP, the P3a was increased in trials preceding correctly detected target trial and the P3b in target trials. Physical training led to a numeric reduction in reaction times to the target but not to changes in the P3a or P3b. As the target detection rate did not change, no improvement of WM was evident. The relaxation training group which exhibited no behavioural changes showed an unexpected reduction in the P3 complex after the training. This may be due to a specific reduction of activity due to relaxation. The no-contact control group showed no changes in behaviour and ERPs which show that repeated testing after 4 months do not markedly change cognition. In sum, a four month multidomain paper and pencil and computerized cognitive training seem to produce the most reliable gains in WM capacity by improved representation of stored items compared to physical or relaxation training and no-contact control group.

## Figures and Tables

**Table 1:** Mean reaction times and mean ratio of missed targets with standard deviations in parentheses in the 0-back and 2-back tasks for the pre- and post-measures and all groups.

	Physical	Cognitive	Relaxation	Control
*Reaction times (ms)*				
Pretest				
0-back	465 (47)	473 (67)	467 (58)	464 (54)
2-back	660 (106)	640 (97)	647 (111)	621 (109)
Posttest				
0-back	467 (61)	464 (50)	467 (59)	471 (50)
2-back	623 (87)	622 (100)	631 (104)	604 (91)
*Ratio of omitted targets (%)*				
Pretest				
0-back	0.2 (1.0)	0 (0.0)	0.2 (1.0)	0.2 (1.0)
2-back	15.7 (14.7)	19.3 (17.8)	20.6 (16.6)	19.9 (23.3)
Posttest				
0-back	0.1 (0.7)	0 (0.0)	0.2 (1.0)	0 (0.0)
2-back	13.9 (12.6)	7.8 (5.7)	15.3 (15.0)	15.2 (14.9)
